# A Novel Leucyl-tRNA Synthetase Inhibitor, MRX-6038, Expresses Anti-Mycobacterium abscessus Activity *In Vitro* and *In Vivo*

**DOI:** 10.1128/aac.00601-22

**Published:** 2022-08-15

**Authors:** Wenye Wu, Siyuan He, Anqi Li, Qi Guo, Zhili Tan, Shicong Liu, Xinghai Wang, Zhemin Zhang, Bing Li, Haiqing Chu

**Affiliations:** a Department of Respiratory and Critical Care Medicine, Shanghai Pulmonary Hospital, School of Medicine, Tongji University, Shanghai, China; b School of Medicine, Tongji University, Shanghai, China; c Shanghai MicuRx Pharmaceutical Co., Ltd., Shanghai, China; d Shanghai Key Laboratory of Tuberculosis, Shanghai Pulmonary Hospital, School of Medicine, Tongji University, Shanghai, China

**Keywords:** MRX-6038, leucyl-tRNA synthetase inhibitor, *Mycobacterium abscessus*, antimicrobial activity, *in vitro*, *in vivo*

## Abstract

Therapeutic options for Mycobacterium abscessus infections are extremely limited, and new drugs are needed. The anti-M. abscessus activity of MRX-6038, a new leucyl-tRNA synthetase inhibitor, was evaluated *in vitro* and *in vivo*. Antimicrobial susceptibility testing was performed on 12 nontuberculosis mycobacteria (NTM) reference strains and 227 clinical NTM isolates. A minimum bactericidal concentration assay was conducted to distinguish the bactericidal versus bacteriostatic activity of MRX-6038. The synergy between MRX-6038 and 12 clinically important antibiotics was determined using a checkerboard assay. The activity of MRX-6038 against M. abscessus residing inside macrophages was also evaluated. Finally, the potency of MRX-6038 *in vivo* was determined in a neutropenic mouse model that mimicked a pulmonary M. abscessus infection. MRX-6038 exhibited high anti-M. abscessus activity against extracellular M. abscessus in culture, with a MIC_50_ of 0.063 mg/L and a MIC_90_ of 0.125 mg/L. Fifty percent of the activity was bactericidal, and fifty percent was bacteriostatic. A synergy between MRX-6038 and clarithromycin or azithromycin was found in 25% of strains. No antagonism was evident between MRX-6038 and 12 antibiotics commonly used to treat NTM infections. MRX-6038 also exhibited activity against intracellular NTM, which caused a significant reduction in the bacterial load in the lungs of M. abscessus-infected neutropenic mice. In conclusion, MRX-6038 was active against M. abscessus *in vitro* and *in vivo*, and it represents a potential candidate for incorporation into strategies by which M. abscessus infections are treated.

## INTRODUCTION

Mycobacterium abscessus, a major pathogenic nontuberculous mycobacterium (NTM) that is abundant in soil and water (1, 2), can cause severe infections in humans (3). Such infections exhibit high rates of morbidity and mortality, especially among patients with chronic lung diseases (4, 5). Previous whole-genome sequencing studies revealed a risk for human-to-human transmission, making M. abscessus infections even more problematic (6). Typically, M. abscessus is resistant to most drugs used to treat tuberculosis, and it is susceptible to only a few antimicrobial agents (7–9). Hence, therapeutic options are limited. Therefore, the development of potent new anti-M. abscessus drugs that avoid the current antibiotic resistance mechanisms is urgent.

Over the past few years, aminoacyl-tRNA synthetases (AARSs) have emerged as new antibacterial targets. AARSs catalyze the acylation reaction between amino acids and tRNA (10, 11). Once AARS activity is inhibited, protein synthesis is terminated, which inhibits bacterial growth (12). The structural divergence between prokaryotic and eukaryotic AARSs indicates the potential for the development of AARS inhibitors as therapeutic targets (12, 13). Leucyl-tRNA synthetase (LeuRS) is a typical class I AARS. Its leucine-specific domain modulates the aminoacylation and proofreading functions of LeuRS, and this is mainly found in prokaryotes (14, 15). Several recent reports suggest that LeuRS inhibitors (e.g., GSK2251052 and GSK656) might be effective in inhibiting M. abscessus growth (16, 17).

MRX-6038 is a novel boron-containing LeuRS inhibitor that was developed by Shanghai MicuRx Pharmaceutical Co., Ltd. Its anti-mycobacterial activity has not been reported previously. The present study was undertaken to clarify and compare the *in vitro* and *in vivo* anti-NTM (especially anti-M. abscessus) activities of MRX-6038 and GSK656, a second LeuRS inhibitor, by evaluating them at the same time. MRX-6038 was effective in inhibiting the growth of M. abscessus *in vitro*, exhibiting MICs comparable to those of GSK656. MRX-6038 expressed bactericidal activity against half of the isolates tested, while GSK656 exhibited only bacteriostatic activity. Furthermore, MRX-6038 effectively inhibited the growth of intracellular organisms in a dose-dependent manner. Its antimicrobial effect was similar to those of GSK656 and linezolid. Importantly, MRX-6038 synergized with clarithromycin and azithromycin and did not antagonize the activity of antibiotics frequently used to treat M. abscessus. Finally, MRX-6038 was comparable to GSK656 and linezolid, and it effectively inhibited the growth of M. abscessus *in vivo* in mouse lungs. As such, this work potentially provides an additional approach by which to treat M. abscessus infections.

## RESULTS

### MRX-6038 is active against M. abscessus.

A total of 194 M. abscessus clinical isolates (148 subsp. *abscessus* and 46 subsp. *massiliense*) and 2 M. abscessus reference strains (ATCC 19977 and CIP 108297) were collected. MRX-6038 and GSK656 exhibited comparable anti-M. abscessus activity toward extracellular M. abscessus in culture. The minimum inhibitory concentration (MIC) ranged from 0.063 to 0.25 mg/L. The MIC_50_ and MIC_90_ for all isolates tested were 0.063 and 0.125 mg/L, respectively (Table 1). The MIC_50_ and MIC_90_ for subsp. *abscessus* and subsp. *massiliense* tested separately were the same. The detailed MIC distribution of all of the clinical isolates is shown in Table S1.

**TABLE 1:** Minimum inhibitory concentrations (MICs) of MRX-6038 and GSK656 for 194 clinical M. abscessus isolates^*a*^

Antimicrobial agent	Subspecies	MIC_50_^*b*^ (mg/L)	MIC_90_^*b*^ (mg/L)	MIC range (mg/L)	No. (%) of strains by MIC (mg/L)			
					0.032	0.063	0.125	0.5
MRX-6038	*Abscessus* (*n* = 148)	0.063	0.125	0.063~0.25	0	118 (79.73)	27 (18.24)	3 (2.03)
	*Massiliense* (*n* = 46)	0.063	0.125	0.063~0.125	0	29 (63.04)	17 (36.96)	0
GSK656	*Abscessus* (*n* = 148)	0.063	0.125	0.032~0.25	4 (2.70)	121 (81.76)	22 (14.86)	1 (0.68)
	*Massiliense* (*n* = 46)	0.063	0.125	0.032~0.125	1 (2.17)	34 (73.91)	11 (23.91)	0

a MIC values were determined using the CLSI document M24-A2 guidelines for aerobic bacteria.

b MIC_50_ and MIC_90_ are the concentrations that inhibit the growth of 50% and 90% of the isolates, respectively.

The susceptibility of 10 NTM reference strains and 14 M. avium, 15 M. intracellulare, and 4 M. fortuitum clinical isolates was also tested. MRX-6038 was active against most NTM reference strains and exhibited greater antibacterial activity against the M. smegmatis, M. fortuitum, M. scrofulaceum, and M. peregrinum reference strains than did GSK656 (MIC ≤ 0.25 mg/L versus MIC ≥ 4 mg/L) (Tables 2 and 3; Table S2). The activity expressed by MRX-6038 toward M. avium and M. intracellulare tended to be significantly greater than that expressed by GSK656 toward the same organisms, that is, MIC ≤ 0.25 mg/L versus MIC > 128 mg/L, respectively (Tables 2 and 3 and Table S2). Four clinical M. fortuitum isolates were sensitive to MRX-6038 but insensitive to GSK656, exhibiting MICs of 0.125 and >8 mg/L, respectively. Taken together, these results indicate that MRX-6038 expresses greater anti-NTM activity than does GSK656.

**TABLE 2:** Antibacterial spectrum of MRX-6038 and GSK656 for NTM reference strains

Strains	MIC (mg/L)	
	MRX-6038	GSK656
M. abscessus ATCC 19977	0.063	0.032 to 0.063
M. massiliense CIP 108297	0.063	0.063
M. smegmatis ATCC 19420	0.125	>8^*a*^
M. fortuitum ATCC 6841	0.125	>8
M. avium ATCC 25291	2	>128
M. intracellulare ATCC 13950	2	>128
M. kansasii ATCC 12478	>8	>8
M. scrofulaceum ATCC 19981	0.25	4
M. gordonae ATCC 14470	0.5	0.5
M. szulgai ATCC 35799	>8	>8
M. xenopi ATCC 19250	>8	>8
M. peregrinum ATCC 700686	0.125	8

a >8 indicates a MIC value greater than the highest MRX-6038 or GSK656 concentration tested.

**TABLE 3:** MICs of MRX-6038 and GSK656 for additional clinical NTM isolates

Species	No. of strains by MIC (mg/L)^*a*^								Total no.	MIC_50_^*b*^ (mg/L)	MIC_90_^*b*^ (mg/L)
	0.125	0.5	1	2	4	8	>8	>128			
M. avium											
MRX-6038	0	2	4	2	4	2	0	-	14	2	8
GSK656	0	0	0	0	0	0	^*d*^	14	14	>128^*c*^	>128^*c*^
M. intracellulare											
MRX-6038	0	0	0	3	10	1	1	-	15	4	8
GSK656	0	0	0	0	0	0	-	15	15	>128^*c*^	>128^*c*^
M. fortuitum											
MRX-6038	4	0	0	0	0	0	0	-	4	-	-
GSK656	0	0	0	0	0	0	4	-	4	-	-

a MIC values were determined using the CLSI guidelines for aerobic bacteria.

b MIC_50_ and MIC_90_ are the concentrations that inhibit the growth of 50% and 90% of the isolates, respectively.

c MIC values greater than the 128 mg/L value that was tested.

d not done.

### MRX-6038 exhibits bactericidal activity.

**FIG 1:**
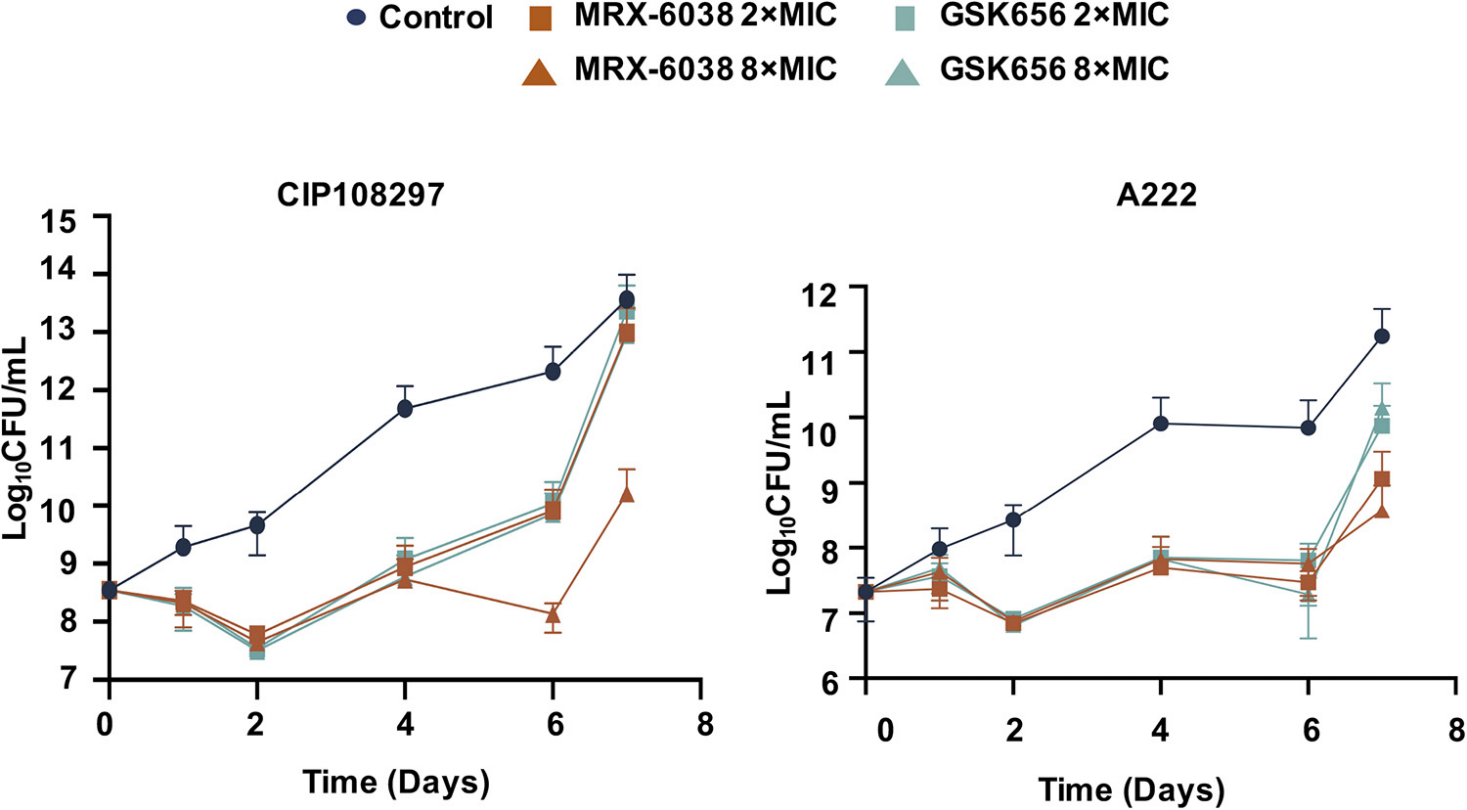
Relative extracellular antimicrobial activities of MRX-6038 and GSK656 *in vitro*. Data are represented as means ± standard deviations of 3 replicates. CIP 108297, subsp. *massiliense* reference strain. A222, subsp. *massiliense* clinical isolate.

The minimum bactericidal concentration (MBC) and MIC values of MRX-6038 and GSK656 for M. abscessus were tested using 20 randomly selected strains (Table S3). The MICs ranged from 0.063 to 0.125 mg/L for both MRX-6038 and GSK656. Higher concentrations were required to kill the isolates tested. Accordingly, the MBC values ranged from 0.25 to 2 mg/mL for MRX-6038 and from 1 to >4 mg/mL for GSK656. The MBC/MIC ratios of MRX-6038 and GSK656 for the isolates tested ranged from 2:16 and 16:>32, respectively (Table 4). For MRX-6038, 10 of the 20 isolates exhibited an MBC/MIC ratio of ≤4, providing evidence of bactericidal activity. GSK656, exhibiting an MBC/MIC ratio of >4, exerted a bacteriostatic effect on all isolates. To further assess the bactericidal activities of MRX-6038 and GSK656, killing kinetics assays (assessed on days 0, 1, 2, 4, 6, and 7) were performed for both drugs at 2 × MIC and 8 × MIC. For MRX-6038 at 2 × MIC, there were significant reductions in both CIP 108297 and A222 from day 1 to day 6, compared to the control (Fig. 1). Treatment with GSK656 at 2 × MIC yielded comparable results. In contrast, treatment with MRX-6038 at 8 × MIC resulted in a greater reduction in M. abscessus colony forming units (CFU) than did a treatment with CIP 108297 with GSK656 at 8 × MIC. Regrowth occurred after 2 to 7 days of incubation with either GSK6038 or GSK656, and this is likely due to a loss of antibiotic potency over time in culture.

**TABLE 4:** MBC/MIC ratios of MRX-6038 and GSK656 for M. abscessus complex

Isolate	Subspecies	MRX-6038	Antibacterial activity	GSK656	Antibacterial activity
A215	*Abscessus*	8	Bacteriostatic	16	Bacteriostatic
A217	*Abscessus*	8	Bacteriostatic	32	Bacteriostatic
A232	*Abscessus*	2	Bactericidal	32	Bacteriostatic
A274	*Abscessus*	16	Bacteriostatic	16	Bacteriostatic
A35	*Abscessus*	4	Bactericidal	32	Bacteriostatic
G73	*Abscessus*	16	Bacteriostatic	>32^*a*^	Bacteriostatic
G164	*Abscessus*	4	Bactericidal	>32	Bacteriostatic
A51	*Abscessus*	4	Bactericidal	>32	Bacteriostatic
A222	*Massiliense*	4	Bactericidal	>32	Bacteriostatic
G74	*Massiliense*	16	Bacteriostatic	>32	Bacteriostatic
A311	*Abscessus*	2	Bactericidal	>32	Bacteriostatic
G104	*Abscessus*	2	Bactericidal	32	Bacteriostatic
A213	*Abscessus*	2	Bactericidal	32	Bacteriostatic
A353	*Abscessus*	2	Bactericidal	>32	Bacteriostatic
A255	*Abscessus*	4	Bactericidal	>32	Bacteriostatic
G87	*Massiliense*	8	Bacteriostatic	16	Bacteriostatic
G182	*Abscessus*	16	Bacteriostatic	32	Bacteriostatic
G110	*Massiliense*	16	Bacteriostatic	>32	Bacteriostatic
A197	*Abscessus*	32	Bacteriostatic	>32	Bacteriostatic
A268	*Abscessus*	16	Bacteriostatic	>32	Bacteriostatic

a A ratio of >32 indicates a MBC value greater than the highest GSK656 concentration tested.

### MRX-6038 is compatible with clinically important anti*-*M. abscessus drugs.

Two M. abscessus reference strains and six clinical isolates were selected at random to investigate the interaction between MRX-6038 and 10 clinically relevant anti*-*M. abscessus drugs, which are enumerated in Table 5. MRX-6038 synergized (fractional inhibitory concentration index [FICI] ≤ 0.5) with clarithromycin and/or azithromycin to inhibit the growth of several *M. massiliense* isolates. No antagonism between MRX-6038 and any of the other drugs was observed, with indifference (FICI = 0.75 to 2) being the most commonly observed interaction.

**TABLE 5:** FICI of MRX-6038 combined with agents commonly used to clinically treat M. abscessus infections

Isolate or reference strain	Subspecies	FICI (MRX-6038 + agent indicated)^*a*^											
		CLA^*b*^	AZM	AMK	BDQ	LNZ	CFZ	IPM	TGC	FOX	MOX	RFB	MRX-1
ATCC 19977	*Abscessus*	1	0.75	1.5	1.5	2	2	1.5	1.5	2	1.5	2	1.5
CIP 108297	*Massiliense*	0.75	0.75	1.5	1	1.5	2	2	1.5	1.5	1.5	1.5	1
A274	*Abscessus*	0.75	0.75	1.5	1.5	1	1.5	1.5	1.5	1.5	2	1.5	1.5
G164	*Abscessus*	0.75	1.5	1.5	1	1.5	2	1.5	1.5	1.25	1.5	1.5	1
A268	*Massiliense*	0.375	0.75	2	1.25	2	2	1.5	1.5	1.5	2	1.5	1.5
G74	*Massiliense*	0.375	0.5	1.5	1	1	1.5	1	1	2	1	1.5	1
A222	*Massiliense*	0.75	0.5	2	1.5	1	1.5	1.5	2	1.5	2	2	1
A217	*Abscessus*	0.75	1	2	1.5	1.5	1.5	2	2	2	2	1.5	1.5

a FICI (fractional inhibitory concentration index) = [(MIC of MRX-6038 in combination/MIC of MRX-6038 alone) + (MIC of the second antibiotic in combination / MIC of the second antibiotic alone)]. Synergy, FICI ≤ 0.5; indifference, FICI greater than 0.5 and ≤4; antagonism, FICI > 4.

b CLA, clarithromycin; AZM, azithromycin; AMK, amikacin; BDQ, bedaquiline; LNZ, linezolid; CFZ, clofazimine; IPM, imipenem; TGC, tigecycline; FOX, cefoxitin; MOX, moxifloxacin; RFB, rifabutin; MRX-1, contezolid.

### MRX-6038 inhibits the growth of intracellular M. abscessus.

**FIG 2:**
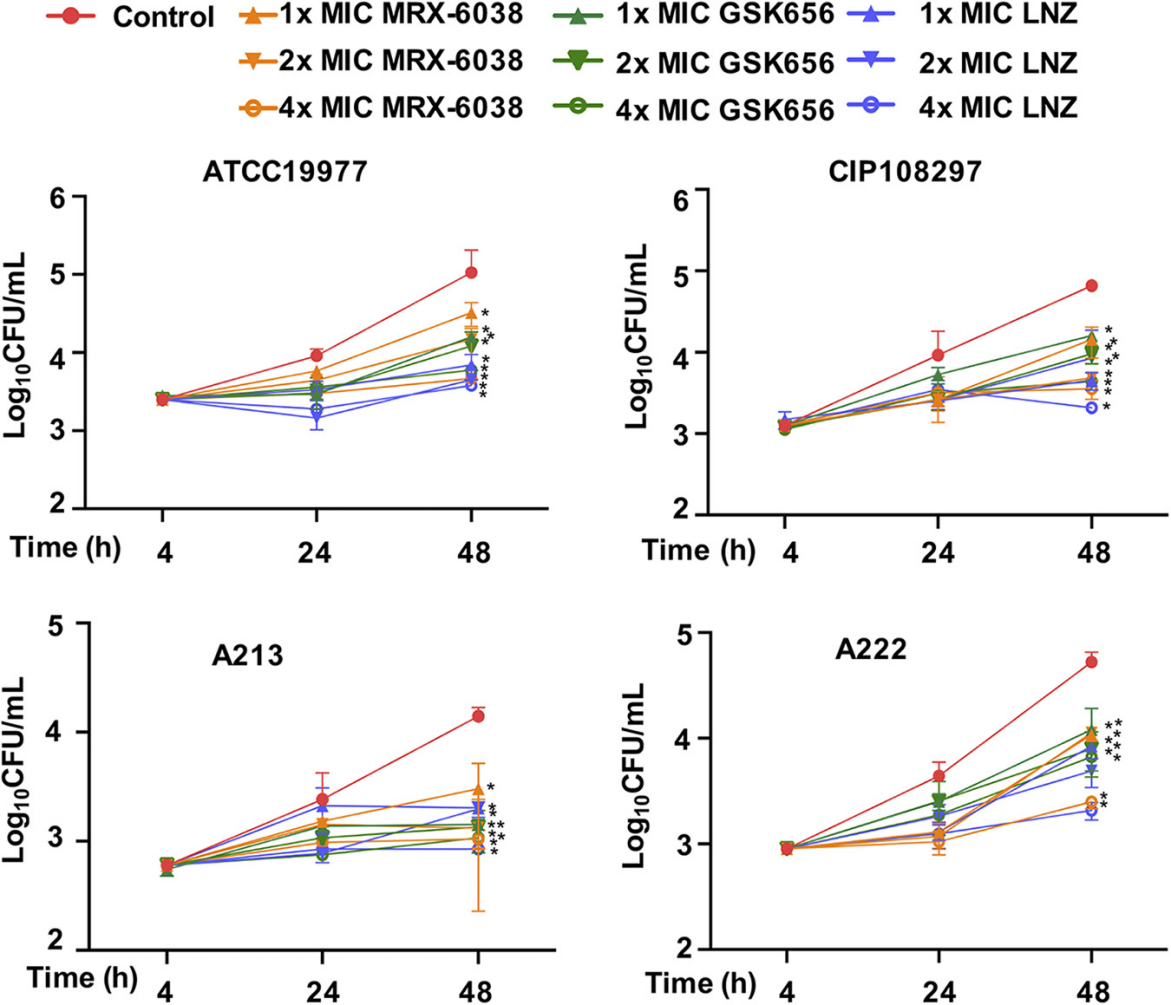
Relative intracellular antimicrobial activities of MRX-6038 and GSK656 *in vitro*. LNZ, linezolid. Data are represented as means ± standard deviations of 3 replicates. Statistically significantly less than the control: *, *P* < 0.05 (Student's *t* test). ATCC 19977, subsp. *abscessus* reference strain. CIP 108297, subsp. *massiliense* reference strain. A213, subsp. *abscessus* clinical isolate. A222, subsp. *massiliense* clinical isolate.

The intracellular antimicrobial activity of MRX-6038 was assessed after M. abscessus replicated for 4 h, 24 h, and 48 h inside J774A.1 macrophages. MRX-6038 at 1 × MIC decreased the number of intracellular mycobacteria present at 48 h (*P* < 0.05) (Fig. 2). The antimicrobial activity of MRX-6038 increased with increasing drug concentration and was comparable to the activity exhibited by GSK656 and linezolid, providing evidence of the efficacy of MRX-6038 in treating intracellular M. abscessus. Notably, neither MRX-6038 nor GSK656 was cytotoxic at 4 × MIC (Fig. S1).

### MRX-6038 inhibits the replication of M. abscessus in a mouse lung infection model.

**FIG 3:**
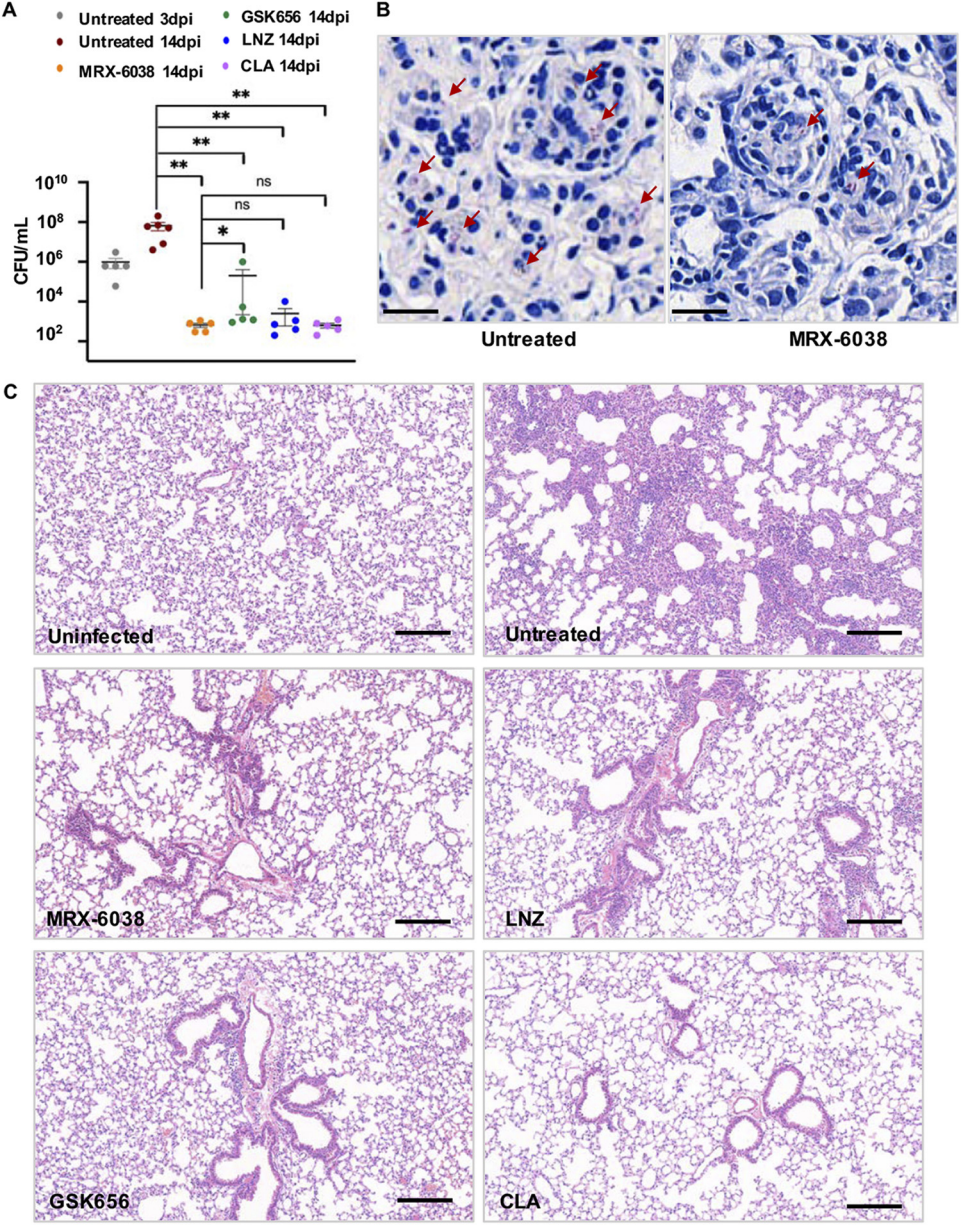
MRX-6038 exhibits anti-M. abscessus activity in a mouse lung infection model. (A) CFU assay. Data are represented as CFUs/lung tissue ± standard deviations with *n* ≥ 5. LNZ, linezolid. CLA, clarithromycin. dpi, days postinfection. Statistical significance: ns, no difference; *, *P* < 0.05; **, *P* < 0.01 (Mann-Whitney *U* test). (B) Acid-fast stained M. abscessus in mouse lung. The scale bar represents 10 μm. (C) Histopathologic assessment of mouse lungs. The lungs were dissected, processed, sectioned, and stained with hematoxylin and eosin. The scale bar represents 100 μm.

Experiments were conducted to evaluate and compare the effects of MRX-6038 and GSK656 on the growth of M. abscessus *in vivo*. Groups of BALB/c mice, inoculated intranasally with M. abscessus CIP 108297 (10^7^ CFU/mouse), were subsequently treated with 10 mg/kg MRX-6038 or GSK656 administered daily s.c., or 100 mg/kg linezolid or 200 mg/kg clarithromycin administered daily by oral gavage. M. abscessus CFU in the lungs were quantified at 2 weeks postinfection. The results showed that MRX-6038 caused a significant reduction in bacteria (~7.8 log_10_ CFU) compared to the untreated group (*P* < 0.01) (Fig. 3A and B). Moreover, MRX-6038 was slightly more potent in clearing M. abscessus than were the other drugs tested. Hematoxylin and eosin (H&E) stained tissue sections demonstrated severe alveolar wall thickening, inflammatory cell infiltration, and diapedesis of the erythrocyte in the lungs of the control group of mice at 2 weeks postinfection (Fig. 3C). In contrast, pathological changes were rare, and lung lesions were negligible in the MRX-6038-treated group. These findings, which provide evidence of the efficacy of MRX-6038 in inhibiting the replication of M. abscessus in a mouse model of pneumonia, are consistent with its intracellular activity and its pharmacokinetic profile, described briefly in the Discussion.

Notably, in the 14 day toxicity study carried out with female SD rats with 40, 80, and 160 mg/kg doses i.v. of MRX-6038, all of the rats in the treated and control groups appeared distress free, exhibited comparable weight gains (ca. 28~34.8 g), and displayed no significant differences in routine blood (Table S5).

### MRX-6038 targets M. abscessus leucyl-tRNA synthetase LeuRS.

MRX-6038 exhibits anti-M. abscessus activity. To confirm the MRX-6038 target, MRX-6038-resistant mutant M. abscessus isolates were selected. Based upon 6 independent selections, a frequency of resistance to MRX-6038 was calculated at 10^9^ CFU. MIC profiling of 10 MRX-6038-resistant mutants revealed a high-level resistance to MRX-6038 but not to clarithromycin (Table 6). According to the data shown in Table 6, the MRX-6038 MIC for these mutants was >0.63, whereas the MIC for the wild-type control was 0.063. Sequencing *leuS* suggested that all resistant isolates had a single missense mutation in the *leuS* editing domain (residues 292 to 502), consistent with the mechanism of leucyl-tRNA synthetase inhibition. The S303L, V417M, and T322I residues were mutated in benzoxaborole-resistant M. abscessus mutants that were previously reported (18, 19). Four other LeuRS missense mutations in the editing domain (i.e., D436E, L418P, Y421H, and D436N) were novel. These results demonstrate that the anti-NTM activity exhibited by MRX-6038 is mediated by targeting the LeuRS editing domain.

**TABLE 6:** Characterization of M. abscessus MRX-6038-resistant mutants

		MIC (mg/L)^*a*^		
Strain	Batch	CLA	MRX-6038	LeuS mutation
M. abscessus ATCC 19977		1	0.063	None
RM1	1	1	>0.63	D436E
RM2	2	1	>1.26	L418P
RM3	2	1	>1.26	L418P
RM4	2	1	>1.26	L418P
RM5	3	0.5	>0.63	Y421H
RM6	3	0.5	>0.63	S303L
RM7	3	0.5	>0.63	D436N
RM8	4	0.5	>1.26	V417M
RM9	4	0.5	>1.26	T322I
RM10	4	0.5	>1.26	V417M

a MIC values are the means obtained in two independent experiments. CLA, clarithromycin.

## DISCUSSION

M. abscessus is a rapidly growing mycobacterium that is highly pathogenic due to its frequent resistance to drug therapy. Infections caused by M. abscessus are difficult to treat with the antibiotics currently available; M. abscessus has been called “a new antibiotic nightmare” (20). Here, the anti-M. abscessus activity of a novel LeuRS inhibitor, MRX-6038, was evaluated and compared to that of another LeuRS inhibitor, GSK656.

The anti-M. abscessus activity exhibited by MRX-6038 toward a large panel of clinical isolates (*n* = 194) retrieved from Shanghai Pulmonary Hospital was initially determined. MRX-6038 was highly active against all isolates (MIC ≤ 0.25 mg/mL) and was equally effective against subsp. *abscessus* and subsp. *massiliense*; the MIC_50_ and MIC_90_ values were 0.063 and 0.125 mg/mL, respectively. Preliminary pharmacokinetic studies indicated that the peak serum concentration in mice administered 5 mg/kg MRX-6038 s.c. exceeded 13 μg/mL (data not shown). This concentration was much higher than the MIC_90_ of MRX-6038 for M. abscessus *in vitro*, indicating that an effective dose could readily be achieved to treat clinical M. abscessus infections. Moreover, there were no signs of toxicity in mice administered a 40, 80, or 160 mg/kg dose of MRX-6038 (Table S5). In similar experiments, a dose of 30 mg/kg GSK656 proved to be safe in a mouse model of M. tuberculosis infection, achieving a peak serum concentration (Cmax) of 17.76 μg/mL (21). The efficacy of 10 mg/kg doses of MRX-6038 and GSK656 were compared in the mouse model of M. abscessus infection described herein. Notably, the optimal dose and formulation of MRX-6038 that provide the maximum therapeutic outcome remain to be determined.

There are numerous reports regarding the anti-M. abscessus activity of LeuRS inhibitors, most of which belong to a new class of LeuRS inhibitors: benzoxaborole (including epetraborole), GSK2251052, EC/11770, and GSK656 (16, 17, 22–24). Epetraborole, a nonhalogenated benzoxaborole, was active against M. abscessus with a MIC ranging from 0.014 to 0.046 mg/L *in vitro* and a 1 log_10_ decrease in bacteria in mice administered a 300 mg/kg dose orally (19, 25). Notably, GSK2251052-related experiments were terminated following the development of resistance in a phase II clinical trial (26). EC/11770 and GSK656 were initially developed to treat M. tuberculosis infections. EC/11770 was active against M. abscessus biofilms *in vitro*. Additionally, a 10 mg/kg dose administered orally inhibited 1 log_10_ bacterial growth in a mouse model of lung infection (18). However, data obtained from GSK656 in previous studies only suggest that it possesses potential anti-M. abscessus activity *in vitro* (17, 26). The study reported herein demonstrates the elevated anti-M. abscessus activity of two LeuRS inhibitors, MRX-6038 and GSK656, for a large number of clinical isolates (17). MRX-6038 appeared to be more active than GSK656, exhibiting a broader anti-NTM spectrum. MRX-6038 expressed greater antibacterial activity toward M. smegmatis, M. fortuitum, M. scrofulaceum, and M. peregrinum reference strains than did GSK656. Its activity toward M. avium and M. intracellulare also tended to be greater. The MBC and MBC/MIC ratio data also support the increased effectivity of MRX-6038 relative to GSK656 and demonstrate its bactericidal activity.

MRX-6038 inhibited the replication of M. abscessus in an *in vitro* intracellular killing assay in a dose-dependent manner; growth was 1.5 log_10_ less in the presence of 0.25 mg/mL MRX-6038 compared to the untreated control. MRX-6038 possessed anti-M. abscessus activity that was comparable to that exhibited by linezolid or clarithromycin (27, 28). A pharmacokinetic study of the blood and lungs of mice administered MRX-6038 supports the potential efficacy of oral delivery for the treatment of M. abscessus infections.

Effective regimens used to treat M. abscessus infections require a combination of antimicrobials, in which macrolides (clarithromycin and azithromycin) are core (22–24). Guo et al. reported 10% synergy and 3.3% antagonism when GSK656 was used in combination with clarithromycin and 20% synergy and 30% antagonism when used in combination with azithromycin (29). In contrast, MRX-6038 exhibited only 25% synergy and 75% indifference when used in combination with clarithromycin or azithromycin in the study reported here. The synergy between MRX-6038 and clarithromycin inhibited the growth of certain *M. massiliense* isolates but not M. abscessus isolates. The *erm*(41), which only existed in *M. massiliense*, was related to the susceptibility of clarithromycin (30). Moreover, MRX-6038 did not antagonize any of the most frequently used anti-M. abscessus drugs. Therefore, MRX-6038 is compatible and could readily be incorporated into current anti-M. abscessus treatment approaches.

AARSs possess several features that provide therapeutic specificity. First, the divergence of sequence, structure, and topology exhibited by pathogenic and human AARSs enable the design of pathogen-specific drugs, such as GSK656 (31). MRX-6038 targets the same AARSs as does GSK656 with greater antibacterial activity. To identify the distinguishing factors, MRX-6038 and GSK656 should be studied and compared, for example, via an electron microscopic analysis of their catalytic domains and their interactions with small molecules. Second, AARSs can target aminoacyl-tRNAs involved in specific pathologies. Halofuginone, for example, inhibits the contribution of prolyl-tRNA synthetase to fibrosis that is closely associated with M. abscessus infection (32, 33). The third feature that makes AARSs attractive for pharmacological intervention is that protein synthesis in not restricted in normal cells, even when AARS activity is severely compromised (34). Such novel AARS features support the potential incorporation of AARS inhibitors into new treatment options for M. abscessus infections.

One limitation inherent in the current study is that all of the tested isolates were obtained from a single hospital, Shanghai Pulmonary Hospital, which is a designated treatment center for tuberculosis and NTM infected patients. Consequently, many of the isolates could be related genetically and epidemiologically. Nonetheless, the study provides a comprehensive description of the *in vitro* and *in vivo* susceptibility of M. abscessus to MRX-6038.

In conclusion, MRX-6038 was highly effective in inhibiting the growth of M. abscessus *in vitro* and *in vivo*, and it was compatible with the antibiotics most frequently used to treat M. abscessus infections. As such, MRX-6038 represents a potential candidate for incorporation into novel therapeutic anti-M. abscessus regimens.

## MATERIALS AND METHODS

### Bacterial strains.

Twelve NTM reference strains and 227 NTM clinical isolates (194 M. abscessus, 14 M. avium, 15 M. intracellulare, and 4 M. fortuitum) were evaluated. The reference strains, M. abscessus subsp. *abscessus* (ATCC 19977), M. smegmatis (ATCC 19420), M. avium (ATCC 25291), M. intracellulare (ATCC 13950), M. kansasii (ATCC 12478), M. fortuitum (ATCC 6841), M. gordonae (ATCC 14470), M. scrofulaceum (ATCC 19981), *M. szulgai* (ATCC 35799), M. xenopi (ATCC 19250), and *M. peregrinum* (ATCC 700686), were purchased from the American Type Culture Collection (VA, USA). M. abscessus subsp. *massiliense* (CIP 108297) was purchased from the Biological Resource Center of Institute Pasteur (Paris, France). The clinical isolates were obtained from the Shanghai Pulmonary Hospital from sputum and bronchoalveolar lavage fluid specimens of patients with NTM lung infections. All M. abscessus isolates were sequenced; the full genome sequence of each isolate was published and is available at DDBJ/ENA/GenBank (BioProject PRJNA488058, PRJNA448987, and PRJNA398137). All strains were grown at 37°C in Middlebrook 7H9 broth supplemented with 10% OADC or on Middlebrook 7H10 plates supplemented with 10% OADC.

### Antimicrobial agents.

MRX-6038 (US2013165411, China National Intellectual Property Administration) and contezolid were provided by MicuRx Pharmaceutical (Shanghai, China). Clarithromycin, azithromycin, amikacin, cefoxitin, imipenem, tigecycline, linezolid, and ciprofloxacin were purchased from Sigma-Aldrich Company (St. Louis, MO, USA). Bedaquiline was purchased from Biopharmaleader (Shanghai, China). GSK656 was purchased from MedChemExpress (Monmouth Junction, NJ, USA). GSK656, clarithromycin, clofazimine, bedaquiline, and rifabutin were solubilized in 10% DMSO, azithromycin was dissolved in absolute ethanol, and the remaining antibiotics were prepared in deionized water. The antibiotics were aliquoted, stored at −20°C and serially diluted just prior to experimental use.

### MIC and MBC determination.

Antibiotic susceptibility was determined by the broth microdilution method, according to Clinical and Laboratory Standards Institute (CLSI) document M24-A2. In brief, individual colonies were picked, grown to logarithmic-phase in M7H9 broth, diluted to McFarland 0.5 with sterile saline, and adjusted to 1 × 10^5^ to 5 × 10^5^ CFU/mL using CAMHB. 100 μL of bacterial suspension was added to each well, except for the peripheral wells, of a 96-well microtiter plate. The peripheral wells were filled with 200 μL sterile water to prevent evaporation during incubation. Antibiotics were serially diluted 1:2 in the plates (working concentrations ranged from 0.008 to 8.0 mg/L for MRX-6038, GSK656, and bedaquiline and from 0.063 to 128 mg/L for the remaining antibiotics tested). The microtiter plates were sealed with Parafilm. Rapid-growing mycobacteria were incubated for 3 to 5 days at 37°C, and slow-growing mycobacteria were incubated for 7 to 10 days at 37°C until the control wells without antibiotics exhibited visible growth. The MIC was defined as the minimum compound concentration at which no visual bacterial growth occurred.

M. abscessus subsp. *abscessus* ATCC 19977, subsp. *massiliense* CIP 108297, and 20 randomly selected clinical M. abscessus isolates were used in assays to determine MBC values. The contents of each microtiter well containing a drug concentration greater than the MIC were evenly suspended on day 4, following the completion of the antimicrobial susceptibility test. 100 μL aliquots obtained from each well were cultured on Middlebrook 7H10 supplemented with 0.2% glycerol and OADC enrichment. CFU were quantified after an additional 5 days of incubation at 37°C. The MBC was defined as the minimum drug concentration that prevented 99.9% bacterial growth, expressed in CFU/mL. An antibiotic was considered bactericidal if the MBC/MIC ratio was ≤4 or bacteriostatic if the ratio was >4 (35).

### Time-kill kinetics assay.

Subsp. *abscessus* ATCC 19977, subsp. *massiliense* CIP 108297, clinical subsp. *abscessus* isolate A213, and clinical subsp. *massiliense* isolate A222 were used to evaluate the potency of the killing kinetics of MRX-6038 and GSK656 against M. abscessus. Tubes (5 mL) of 7H9 broth containing MRX-6038 or GSK656 at 1 × MIC or 4 × MIC were inoculated with 5 × 10^5^ CFU of bacteria growing exponentially. The tubes were incubated for 7 days at 37°C. The CFU were quantified by plating serial dilutions of bacteria on 7H10 agar plates on days 0, 1, 2, 4, 6, and 7 of incubation.

### Drug synergy.

The synergy between MRX5 and clarithromycin, azithromycin, amikacin, linezolid, clofazimine, imipenem, tigecycline, bedaquiline, cefoxitin, rifabutin, moxifloxacin, or contezolid was assessed *in vitro* using the previously described broth microdilution checkerboard titration technique (36). Two M. abscessus reference strains (subsp. *abscessus* ATCC 19977 and subsp. *massiliense* CIP 108297) and six clinical isolates (three subsp. *abscessus* and three subsp. *massiliense*) were used. Synergy test results were interpreted based upon the FICI, which was calculated using the following formula: FICI = (MIC of antibiotic A in combination/MIC of antibiotic A alone) + (MIC of MRX-6038 in combination / MIC of MRX-6038 alone). Drug interactions were classified as: synergistic (FICI ≤ 0.5), indifferent (0.5 < FICI ≤ 4), or antagonistic (FICI > 4.0).

### Cytotoxicity assay.

J774A.1 cells were suspended in fresh RPMI 1640 medium containing 10% FBS and 4 × MIC of MRX-6038 or GSK656. The control cells were suspended in medium with 10% FBS only. Cells were incubated at 37°C in 5% CO_2_ for 4, 24, or 48 h. The supernates were carefully collected at each time point after being centrifuged at 1,000 rpm for 5 min and analyzed by the CytoTox 96 Non-Radioactive Cytotoxicity Assay (Promega, Madison, WI, USA), which quantifies lactate dehydrogenase (LDH), a stable cytosolic enzyme that is released upon cell lysis. The cytotoxicity of the antimicrobial agents was assessed by comparing the LDH (OD_490_) released by the experimental and control groups (37). No cytotoxicity was defined as no significant difference being observed between the control and experimental groups.

In the 14 day toxicity study in rats, female SD rats were intravenously administered at the doses of 40, 80, and 160 mg/kg formulated with dextrose 5% in water. After 14 days of administration, the cytotoxicity of the antimicrobial agents was assessed using the body weight, blood biochemical, and blood routine indices of all of the rats.

### Intracellular killing assay.

The macrophage cell line, J774A.1, is frequently used to examine the factors that affect the intracellular replication of mycobacterium species (38). J774A.1 cells were infected with M. abscessus (multiplicity of infection = 10) and suspended in RPMI 1640 medium supplemented with 10% fetal bovine serum (FBS). After 4 h of incubation at 37°C in 5% CO_2_, fresh RPMI 1640 medium containing 10% FBS and amikacin at 50 μg/mL were added (39, 40). After 2 additional hours of incubation at 37°C, the cells were washed three times with warm phosphate-buffered saline to eliminate the viable extracellular organisms that remained. Serial dilutions of the supernates collected after the final wash were cultured on agar plates, and the CFU were counted to ensure that the residual extracellular bacteria were completely removed. Fresh RPMI 1640 medium with 10% FBS and MRX-6038, GSK656, or linezolid at 0.5 × MIC, 1 × MIC, 2 × MIC, and 4 × MIC was then added. Infected control cells were treated with medium and 10% FBS alone. The cells were then lysed with 0.05% sodium dodecyl sulfate after 4, 24, or 48 hours of infection, and the CFU were quantified by plating serial dilutions of lysates on 7H10 agar plates. Cell viability was evaluated by trypan blue exclusion before and after infection or drug treatment at each time point.

### Efficacy of MRX-6038 in M. abscessus infected neutropenic mice.

All animal experiments were approved by the Institutional Animals Ethics Committee of Shanghai Pulmonary Hospital (Protocol No. K22-031Y). Studies were conducted using an immunocompromised mouse model that was modified to mimic a respiratory infection. Briefly, 6-week-old male BALB/c mice (20 to 22 g) were rendered neutropenic via the intraperitoneal injection of 150 mg/kg cyclophosphamide on days 4 and 1 prior to infection. The mice were then infected intranasally with M. abscessus CIP 108297 (1 × 10^7^ CFU/mouse). On day 3 postinfection, five mice were euthanized, and the CFU/lung values were determined to evaluate the model’s efficacy. The remaining 26 infected mice were divided randomly into five groups: group 1 (control, PBS with 0.5% methylcellulose–0.1% Tween 80 administered s.c. daily for 2 weeks), group 2 (10 mg/kg MRX-6038 in PBS with 0.5% methylcellulose–0.1% Tween 80 administered s.c. daily for 2 weeks), group 3 (10 mg/kg GSK656 administered s.c. daily for 2 weeks), group 4 (100 mg/kg linezolid administered by oral gavage daily for 2 weeks), and group 5 (200 mg/kg clarithromycin administered by oral gavage daily for 2 weeks). The dosages of linezolid and clarithromycin were selected based upon a prior report authored by Kim et al. (20). The same dose of MRX-6038 and GSK656 was chosen to facilitate comparison. The mice were euthanized on day 14 postinfection. For a histopathological examination, the upper lobes of the right lungs of mice from each group were dissected, fixed with 4% paraformaldehyde for 24 h, and sectioned. The tissue sections were subjected to H&E and acid-fast staining. The remaining portions of the lungs were homogenized, and 10-fold serial dilutions of the homogenates were plated on M7H10 agar supplemented with 10% Middlebrook OADC enrichment. The bacterial content in the lungs was estimated from the number of colonies that grew on plates incubated for 5 days at 37°C.

### Selection of spontaneous resistant mutants.

M. abscessus colonies were grown in broth to an OD_600_ ranging from 0.1 to 0.4, centrifuged, and resuspended at OD_600_ = 10. 5 mL culture aliquots were kept as references for sequencing. 100 μL (~1 × 10^9^ CFU/μL) were plated on solid media containing MRX-6038 at 10 × , 20 × , or 40 × MIC_90_. Plates were incubated for 7 days at 37°C. The drug-resistant colonies were picked, resuspended in broth, and confirmed as resistant via restreaking on agar containing the same concentration of drug. Genomic DNA was extracted using a DNA extraction kit (Magen, China). Sanger sequencing of the *leuS* (*MAB_4923*) genomic region was performed by Qingke Company (Shanghai, China), using the following four primers: (leuS-UpStrm-Fwd: 5′-GTCCCGAAGTTAATAACCGC-3′; leuS-Int-Fwd: 5′-GACGCAGTGGATTTTCCTAC-3′; leuS-Int-Rev: 5′-AGGCTCTTTCCGATCTTCCC-3′; and leuS-DwnStrm-Rev: 5′-AGAACTCACCGAACATGAAG-3′) (18). All spontaneous resistant mutants were determined to possess a missense mutation in the editing domain of M. abscessus LeuRS.

### Statistical analysis.

Statistical differences between study groups were determined using the Mann-Whitney *U*-test and Student’s *t* test; a *P* value of <0.05 was considered to be indicative of a statistically significant result. Computations were performed using GraphPad Prism 8 (GraphPad Software, San Diego, CA).

### Ethical approval.

The use of mice in this study was approved by the Institutional Animals Ethics Committee of Shanghai Pulmonary Hospital (Protocol No. K22-031Y).

## References

[1] Benwill JL, Wallace RJ, Jr. 2014. *Mycobacterium abscessus*: challenges in diagnosis and treatment. Curr Opin Infect Dis 27:506–510. 10.1097/QCO.0000000000000104. 25268925

[2] Johansen MD, Herrmann JL, Kremer L. 2020. Non-tuberculous mycobacteria and the rise of *Mycobacterium abscessus*. Nat Rev Microbiol 18:392–407. 10.1038/s41579-020-0331-1. 32086501

[3] Cowman S, van Ingen J, Griffith DE, Loebinger MR. 2019. Non-tuberculous mycobacterial pulmonary disease. Eur Respir J 54:1900250. 10.1183/13993003.00250-2019. 31221809

[4] Bryant JM, Brown KP, Burbaud S, Everall I, Belardinelli JM, Rodriguez-Rincon D, Grogono DM, Peterson CM, Verma D, Evans IE, Ruis C, Weimann A, Arora D, Malhotra S, Bannerman B, Passemar C, Templeton K, MacGregor G, Jiwa K, Fisher AJ, Blundell TL, Ordway DJ, Jackson M, Parkhill J, Floto RA. 2021. Stepwise pathogenic evolution of *Mycobacterium abscessus*. Science 372. 10.1126/science.abb8699. PMC7611193 33926925

[5] Hughes DA, Bokobza I, Carr SB. 2021. Eradication success for non-tuberculous mycobacteria in children with cystic fibrosis. Eur Respir J 57:2003636. 10.1183/13993003.03636-2020. 33542059 PMC8280568

[6] Bryant JM, Grogono DM, Greaves D, Foweraker J, Roddick I, Inns T, Reacher M, Haworth CS, Curran MD, Harris SR, Peacock SJ, Parkhill J, Floto RA. 2013. Whole-genome sequencing to identify transmission of *Mycobacterium abscessus* between patients with cystic fibrosis: a retrospective cohort study. Lancet 381:1551–1560. 10.1016/S0140-6736(13)60632-7. 23541540 PMC3664974

[7] Bryant JM, Grogono DM, Rodriguez-Rincon D, Everall I, Brown KP, Moreno P, Verma D, Hill E, Drijkoningen J, Gilligan P, Esther CR, Noone PG, Giddings O, Bell SC, Thomson R, Wainwright CE, Coulter C, Pandey S, Wood ME, Stockwell RE, Ramsay KA, Sherrard LJ, Kidd TJ, Jabbour N, Johnson GR, Knibbs LD, Morawska L, Sly PD, Jones A, Bilton D, Laurenson I, Ruddy M, Bourke S, Bowler IC, Chapman SJ, Clayton A, Cullen M, Daniels T, Dempsey O, Denton M, Desai M, Drew RJ, Edenborough F, Evans J, Folb J, Humphrey H, Isalska B, Jensen-Fangel S, Jonsson B, Jones AM, et al. 2016. Emergence and spread of a human-transmissible multidrug-resistant nontuberculous mycobacterium. Science 354:751–757. 10.1126/science.aaf8156. 27846606 PMC5142603

[8] Tortoli E, Kohl TA, Trovato A, Baldan R, Campana S, Cariani L, Colombo C, Costa D, Cristadoro S, Di Serio MC, Manca A, Pizzamiglio G, Rancoita PMV, Rossolini GM, Taccetti G, Teri A, Niemann S, Cirillo DM. 2017. *Mycobacterium abscessus* in patients with cystic fibrosis: low impact of inter-human transmission in Italy. Eur Respir J 50:1602525. 10.1183/13993003.02525-2016. 28705942

[9] Griffith DE. 2019. *Mycobacterium abscessus* and antibiotic resistance: same as it ever was. Clin Infect Dis 69:1687–1689. 10.1093/cid/ciz071. 30689764

[10] Dewan V, Reader J, Forsyth KM. 2014. Role of aminoacyl-tRNA synthetases in infectious diseases and targets for therapeutic development. Top Curr Chem 344:293–329. 10.1007/128_2013_425. 23666077

[11] Lee EY, Kim S, Kim MH. 2018. Aminoacyl-tRNA synthetases, therapeutic targets for infectious diseases. Biochem Pharmacol 154:424–434. 10.1016/j.bcp.2018.06.009. 29890143 PMC7092877

[12] Woese CR, Olsen GJ, Ibba M, Soll D. 2000. Aminoacyl-tRNA synthetases, the genetic code, and the evolutionary process. Microbiol Mol Biol Rev 64:202–236. 10.1007/s00239-021-10029-x. 10704480 PMC98992

[13] Melnikov SV, Soll D. 2019. Aminoacyl-tRNA Synthetases and tRNAs for an expanded genetic code: what makes them orthogonal? Int J Mol Sci 20:1929. 10.3390/ijms20081929. PMC6515474 31010123

[14] Yan W, Tan M, Eriani G, Wang ED. 2013. Leucine-specific domain modulates the aminoacylation and proofreading functional cycle of bacterial leucyl-tRNA synthetase. Nucleic Acids Res 41:4988–4998. 10.1093/nar/gkt185. 23525458 PMC3643597

[15] Huang Q, Zhou XL, Hu QH, Lei HY, Fang ZP, Yao P, Wang ED. 2014. A bridge between the aminoacylation and editing domains of leucyl-tRNA synthetase is crucial for its synthetic activity. RNA 20:1440–1450. 10.1261/rna.044404.114. 25051973 PMC4138327

[16] Kim T, Hanh BT, Heo B, Quang N, Park Y, Shin J, Jeon S, Park JW, Samby K, Jang J. 2021. A screening of the MMV Pandemic Response Box reveals epetraborole as a new potent inhibitor against *Mycobacterium abscessus*. Int J Mol Sci 22:5936. 10.3390/ijms22115936. 34073006 PMC8199016

[17] Dong W, Li S, Wen S, Jing W, Shi J, Ma Y, Huo F, Gao F, Pang Y, Lu J. 2020. *In Vitro* susceptibility testing of GSK656 against *Mycobacterium* species. Antimicrob Agents Chemother 64:e01577-19. 10.1128/AAC.01577-19. 31791947 PMC6985724

[18] Ganapathy US, Del Rio RG, Cacho-Izquierdo M, Ortega F, Lelièvre J, Barros-Aguirre D, Lindman M, Dartois V, Gengenbacher M, Dick T. 2021. A leucyl-tRNA aynthetase inhibitor with broad-spectrum anti-mycobacterial activity. Antimicrob Agents Chemother 65. 10.1128/AAC.02420-20. PMC8092876 33558292

[19] Ganapathy US, Gengenbacher M, Dick T. 2021. Epetraborole is active against *Mycobacterium abscessus*. Antimicrob Agents Chemother 65:e0115621. 10.1128/AAC.01156-21. 34280020 PMC8448144

[20] Nessar R, Cambau E, Reyrat JM, Murray A, Gicquel B. 2012. *Mycobacterium abscessus*: a new antibiotic nightmare. J Antimicrob Chemother 67:810–818. 10.3390/microorganisms9030596. 22290346

[21] Palencia A, Li X, Bu W, Choi W, Ding CZ, Easom EE, Feng L, Hernandez V, Houston P, Liu L, Meewan M, Mohan M, Rock FL, Sexton H, Zhang S, Zhou Y, Wan B, Wang Y, Franzblau SG, Woolhiser L, Gruppo V, Lenaerts AJ, O'Malley T, Parish T, Cooper CB, Waters MG, Ma Z, Ioerger TR, Sacchettini JC, Rullas J, Angulo-Barturen I, Pérez-Herrán E, Mendoza A, Barros D, Cusack S, Plattner JJ, Alley MR. 2016. Discovery of novel oral protein synthesis inhibitors of *Mycobacterium tuberculosis* that target leucyl-tRNA synthetase. Antimicrob Agents Chemother 60:6271–6280. 10.1128/AAC.01339-16. 27503647 PMC5038265

[22] Haworth CS, Banks J, Capstick T, Fisher AJ, Gorsuch T, Laurenson IF, Leitch A, Loebinger MR, Milburn HJ, Nightingale M, Ormerod P, Shingadia D, Smith D, Whitehead N, Wilson R, Floto RA. 2017. British Thoracic Society guidelines for the management of non-tuberculous mycobacterial pulmonary disease (NTM-PD). Thorax 72:ii1–ii64. 10.1136/thoraxjnl-2017-210927. 29054853

[23] Kwak N, Dalcolmo MP, Daley CL, Eather G, Gayoso R, Hasegawa N, Jhun BW, Koh WJ, Namkoong H, Park J, Thomson R, van Ingen J, Zweijpfenning SMH, Yim JJ. 2019. *Mycobacterium abscessus* pulmonary disease: individual patient data meta-analysis. Eur Respir J 54:1801991. 10.1183/13993003.01991-2018. 30880280

[24] Chen J, Zhao L, Mao Y, Ye M, Guo Q, Zhang Y, Xu L, Zhang Z, Li B, Chu H. 2019. Clinical efficacy and adverse effects of antibiotics used to treat *Mycobacterium abscessus* pulmonary disease. Front Microbiol 10:1977. 10.3389/fmicb.2019.01977. 31507579 PMC6716072

[25] Sullivan JR, Lupien A, Kalthoff E, Hamela C, Taylor L, Munro KA, Schmeing TM, Kremer L, Behr MA. 2021. Efficacy of epetraborole against *Mycobacterium abscessus* is increased with norvaline. PLoS Pathog 17:e1009965. 10.1371/journal.ppat.1009965. 34637487 PMC8535176

[26] O'Dwyer K, Spivak AT, Ingraham K, Min S, Holmes DJ, Jakielaszek C, Rittenhouse S, Kwan AL, Livi GP, Sathe G, Thomas E, Van Horn S, Miller LA, Twynholm M, Tomayko J, Dalessandro M, Caltabiano M, Scangarella-Oman NE, Brown JR. 2015. Bacterial resistance to leucyl-tRNA synthetase inhibitor GSK2251052 develops during treatment of complicated urinary tract infections. Antimicrob Agents Chemother 59:289–298. 10.1128/AAC.03774-14. 25348524 PMC4291364

[27] Novosad SA, Beekmann SE, Polgreen PM, Mackey K, Winthrop KL, Team MAS, M. abscessus Study Team. 2016. Treatment of *Mycobacterium abscessus* infection. Emerging Infectious Diseases 22:511–514. 10.3201/eid2203.150828. 26890211 PMC4766900

[28] Bastian S, Veziris N, Roux AL, Brossier F, Gaillard JL, Jarlier V, Cambau E. 2011. Assessment of clarithromycin susceptibility in strains belonging to the *Mycobacterium abscessus* group by erm(41) and rrl sequencing. Antimicrob Agents Chemother 55:775–781. 10.1128/AAC.00861-10. 21135185 PMC3028756

[29] Guo H, Chen L, Huo F, Pang Y, Li S. 2021. The study of in vitro synergistic activities of GSK656 with clarithromycin or azithromycin against *Mycobacterium abscessus*. Chinese J Antituberculosis 43:153–158.

[30] Li B, Yang S, Chu H, Zhang Z, Liu W, Luo L, Ma W, Xu X. 2017. Relationship between antibiotic susceptibility and genotype in *Mycobacterium abscessus* clinical isolates. Front Microbiol 8:1739. 10.3389/fmicb.2017.01739. 28959242 PMC5603792

[31] Li X, Hernandez V, Rock FL, Choi W, Mak YSL, Mohan M, Mao W, Zhou Y, Easom EE, Plattner JJ, Zou W, Perez-Herran E, Giordano I, Mendoza-sLosana A, Alemparte C, Rullas J, Angulo-Barturen I, Crouch S, Ortega F, Barros D, Alley MRK. 2017. Discovery of a potent and specific *M. tuberculosis* leucyl-tRNA synthetase inhibitor: (S)-3-(aminomethyl)-4-chloro-7–(2-hydroxyethoxy)benzo[c][1,2]oxaborol-1(3H)-ol (GSK656). J Med Chem 60:8011–8026. 10.1021/acs.jmedchem.7b00631. 28953378

[32] Keller TL, Zocco D, Sundrud MS, Hendrick M, Edenius M, Yum J, Kim YJ, Lee HK, Cortese JF, Wirth DF, Dignam JD, Rao A, Yeo CY, Mazitschek R, Whitman M. 2012. Halofuginone and other febrifugine derivatives inhibit prolyl-tRNA synthetase. Nat Chem Biol 8:311–317. 10.1038/nchembio.790. 22327401 PMC3281520

[33] Degiacomi G, Sammartino JC, Chiarelli LR, Riabova O, Makarov V, Pasca MR. 2019. *Mycobacterium abscessus*, an emerging and worrisome pathogen among cystic fibrosis patients. Int J Mol Sci 20:5868. 10.3390/ijms20235868. PMC6928860 31766758

[34] Xu Z, Lo WS, Beck DB, Schuch LA, Oláhová M, Kopajtich R, Chong YE, Alston CL, Seidl E, Zhai L, Lau CF, Timchak D, LeDuc CA, Borczuk AC, Teich AF, Juusola J, Sofeso C, Müller C, Pierre G, Hilliard T, Turnpenny PD, Wagner M, Kappler M, Brasch F, Bouffard JP, Nangle LA, Yang XL, Zhang M, Taylor RW, Prokisch H, Griese M, Chung WK, Schimmel P. 2018. Bi-allelic mutations in Phe-tRNA synthetase associated with a multi-system pulmonary disease support non-translational function. Am J Hum Genet 103:100–114. 10.1016/j.ajhg.2018.06.006. 29979980 PMC6035289

[35] Clinical & Laboratory Standards Institute W. 1999. M26-A: Methods for Determining Bactericidal Activity of Antimicrobial Agent. CLSI, PA.

[36] Kaushik A, Ammerman NC, Tasneen R, Story-Roller E, Dooley KE, Dorman SE, Nuermberger EL, Lamichhane G. 2017. *In vitro* and *in vivo* activity of biapenem against drug-susceptible and rifampicin-resistant *Mycobacterium tuberculosis*. J Antimicrob Chemother 72:2320–2325. 10.1093/jac/dkx152. 28575382 PMC5890701

[37] Riss T, Niles A, Moravec R, Karassina N, Vidugiriene J. 2004. Cytotoxicity assays: in vitro methods to measure dead cells. *In* Markossian S, Grossman A, Brimacombe K, Arkin M, Auld D, Austin CP, Baell J, Chung TDY, Coussens NP, Dahlin JL, Devanarayan V, Foley TL, Glicksman M, Hall MD, Haas JV, Hoare SRJ, Inglese J, Iversen PW, Kales SC, Lal-Nag M, Li Z, McGee J, McManus O, Riss T, Saradjian P, Sittampalam GS, Tarselli M, Trask OJ, Jr., Wang Y, Weidner JR, Wildey MJ, Wilson K, Xia M, Xu X (ed), Assay Guidance Manual. Eli Lilly & Company and the National Center for Advancing Translational Sciences, Bethesda (MD). 31070879

[38] Kerkez I, Tulkens PM, Tenson T, Van Bambeke F, Putrinš M. 2021. Uropathogenic *Escherichia coli* shows antibiotic tolerance and growth heterogeneity in an in vitro model of intracellular infection. Antimicrob Agents Chemother 65:e0146821. 10.1128/AAC.01468-21. 34570646 PMC8597737

[39] Kim BR, Kim BJ, Kook YH, Kim BJ. 2020. *Mycobacterium abscessus* infection leads to enhanced production of type 1 interferon and NLRP3 inflammasome activation in murine macrophages via mitochondrial oxidative stress. PLoS Pathog 16:e1008294. 10.1371/journal.ppat.1008294. 32210476 PMC7094820

[40] Ruth MM, Koeken V, Pennings LJ, Svensson EM, Wertheim HFL, Hoefsloot W, van Ingen J. 2020. Is there a role for tedizolid in the treatment of non-tuberculous mycobacterial disease? J Antimicrob Chemother 75:609–617. 10.1093/jac/dkz511. 31886864 PMC7021090

