# Supplementary material for: A Novel Leucyl-tRNA Synthetase Inhibitor, MRX-6038, Expresses Anti-Mycobacterium abscessus Activity In Vitro and In Vivo
Source: Antimicrob Agents Chemother. 2022 Aug 15;66(9):e00601-22. doi: 10.1128/aac.00601-22 (PMC9487484; doi:10.1128/aac.00601-22)
Supplement: Supplemental file 1 — Tables S1 to S5 and Fig. S1. Download aac.00601-22-s0001.pdf, PDF file, 0.3 MB [file aac.00601-22-s0001.pdf]

Supplementary Materials

Wu *et al.* A Novel leucyl-tRNA synthetase inhibitor, MRX-6038, expresses anti-*Mycobacterium abscessus* activity *in vitro* and *in vivo*

Table S1. MICs of MRX-6038 and GSK656 for 194 *M. abscessus* clinical isolates

Isolate	MICs of MRX-6038 (mg/L)	MICs of GSK656 (mg/L)	Subspecies
A8	0.063	0.063	*subsp. abscessus*
A10	0.063	0.063	*subsp. abscessus*
A25	0.063	0.063	*subsp. abscessus*
A35	0.063	0.063	*subsp. abscessus*
A38	0.063	0.063	*subsp. abscessus*
A39	0.063	0.063	*subsp. massiliense*
A40	0.063	0.063	*subsp. abscessus*
A49	0.063	0.063	*subsp. abscessus*
A51	0.063	0.063	*subsp. abscessus*
A54	0.063	0.063	*subsp. abscessus*
A58	0.125	0.125	*subsp. abscessus*
A59	0.063	0.063	*subsp. abscessus*
A63	0.125	0.063	*subsp. massiliense*
A69	0.063	0.063	*subsp. abscessus*
A73	0.063	0.032	*subsp. abscessus*
A79	0.063	0.032	*subsp. abscessus*
A126	0.125	0.125	*subsp. abscessus*
A137	0.063	0.063	*subsp. abscessus*
A173	0.063	0.032	*subsp. massiliense*
A175	0.063	0.063	*subsp. abscessus*
A176	0.063	0.063	*subsp. abscessus*
A182	0.063	0.063	*subsp. abscessus*
A183	0.063	0.063	*subsp. abscessus*
A186	0.063	0.063	*subsp. massiliense*
A189	0.125	0.125	*subsp. abscessus*
A197	0.063	0.063	*subsp. abscessus*
A205	0.063	0.063	*subsp. massiliense*
A213	0.125	0.063	*subsp. abscessus*
A215	0.063	0.063	*subsp. abscessus*
A217	0.063	0.063	*subsp. abscessus*
A218	0.125	0.125	*subsp. abscessus*
A222	0.063	0.063	*subsp. massiliense*
A228	0.125	0.125	*subsp. massiliense*
A232	0.063	0.063	*subsp. abscessus*
A233	0.063	0.063	*subsp. abscessus*
A243	0.125	0.125	*subsp. abscessus*

A244	0.063	0.063	*subsp. abscessus*
A247	0.125	0.125	*subsp. massiliense*
A249	0.063	0.032	*subsp. abscessus*
A254	0.063	0.063	*subsp. massiliense*
A255	0.063	0.063	*subsp. abscessus*
A266	0.063	0.063	*subsp. abscessus*
A267	0.063	0.063	*subsp. massiliense*
A268	0.125	0.125	*subsp. massiliense*
A274	0.125	0.125	*subsp. abscessus*
A289	0.125	0.063	*subsp. massiliense*
A295	0.125	0.125	*subsp. massiliense*
A297	0.063	0.063	*subsp. abscessus*
A305	0.063	0.063	*subsp. abscessus*
A311	0.063	0.063	*subsp. abscessus*
A312	0.063	0.063	*subsp. abscessus*
A315	0.063	0.063	*subsp. abscessus*
A317	0.063	0.063	*subsp. abscessus*
A321	0.125	0.125	*subsp. abscessus*
A323	0.063	0.063	*subsp. massiliense*
A329	0.125	0.063	*subsp. abscessus*
A330	0.063	0.063	*subsp. abscessus*
A337	0.063	0.063	*subsp. abscessus*
A350	0.063	0.063	*subsp. abscessus*
A353	0.125	0.125	*subsp. abscessus*
129	0.063	0.063	*subsp. abscessus*
G70	0.125	0.125	*subsp. abscessus*
3	0.063	0.063	*subsp. abscessus*
G72	0.063	0.063	*subsp. abscessus*
G73	0.063	0.063	*subsp. abscessus*
G74	0.125	0.125	*subsp. massiliense*
G75	0.125	0.063	*subsp. massiliense*
G76	0.063	0.063	*subsp. abscessus*
G77	0.063	0.063	*subsp. massiliense*
G78	0.063	0.063	*subsp. abscessus*
G79	0.25	0.125	*subsp. abscessus*
2	0.063	0.063	*subsp. abscessus*
G82	0.063	0.063	*subsp. abscessus*
G84	0.125	0.125	*subsp. abscessus*
G85	0.063	0.063	*subsp. massiliense*
G86	0.125	0.125	*subsp. abscessus*
G87	0.063	0.063	*subsp. massiliense*
G88	0.125	0.125	*subsp. massiliense*
G89	0.063	0.063	*subsp. abscessus*
G90	0.063	0.063	*subsp. abscessus*
G91	0.063	0.063	*subsp. abscessus*

G93	0.063	0.125	*subsp. abscessus*
G94	0.063	0.063	*subsp. abscessus*
G95	0.125	0.125	*subsp. massiliense*
G98	0.063	0.063	*subsp. massiliense*
G99	0.125	0.063	*subsp. abscessus*
289	0.063	0.063	*subsp. abscessus*
G101	0.125	0.125	*subsp. massiliense*
G102	0.063	0.063	*subsp. abscessus*
G103	0.063	0.063	*subsp. abscessus*
G104	0.125	0.063	*subsp. abscessus*
G105	0.063	0.063	*subsp. massiliense*
G106	0.063	0.063	*subsp. abscessus*
G107	0.063	0.063	*subsp. massiliense*
G108	0.063	0.063	*subsp. massiliense*
G109	0.25	0.25	*subsp. abscessus*
G110	0.063	0.063	*subsp. massiliense*
G111	0.063	0.063	*subsp. abscessus*
G112	0.125	0.125	*subsp. abscessus*
G113	0.063	0.063	*subsp. abscessus*
G114	0.063	0.063	*subsp. abscessus*
G115	0.063	0.063	*subsp. abscessus*
G116	0.063	0.063	*subsp. massiliense*
G117	0.063	0.063	*subsp. abscessus*
G118	0.125	0.125	*subsp. abscessus*
G119	0.063	0.063	*subsp. abscessus*
G120	0.063	0.063	*subsp. abscessus*
G121	0.063	0.063	*subsp. abscessus*
G122	0.063	0.063	*subsp. abscessus*
G123	0.063	0.063	*subsp. abscessus*
G124	0.063	0.063	*subsp. massiliense*
G125	0.063	0.063	*subsp. abscessus*
G126	0.125	0.063	*subsp. massiliense*
G127	0.063	0.063	*subsp. abscessus*
G128	0.063	0.063	*subsp. abscessus*
G129	0.125	0.125	*subsp. abscessus*
G132	0.125	0.125	*subsp. abscessus*
G133	0.063	0.063	*subsp. abscessus*
G134	0.063	0.063	*subsp. abscessus*
G135	0.063	0.063	*subsp. massiliense*
G136	0.063	0.063	*subsp. abscessus*
G137	0.063	0.063	*subsp. massiliense*
G138	0.063	0.063	*subsp. massiliense*
G139	0.063	0.063	*subsp. abscessus*
G140	0.063	0.063	*subsp. abscessus*
G141	0.063	0.063	*subsp. massiliense*

G142	0.063	0.063	*subsp. abscessus*
G143	0.063	0.063	*subsp. abscessus*
G144	0.063	0.063	*subsp. abscessus*
G145	0.063	0.063	*subsp. abscessus*
G146	0.125	0.125	*subsp. abscessus*
G147	0.125	0.125	*subsp. massiliense*
G148	0.125	0.063	*subsp. abscessus*
G149	0.125	0.125	*subsp. abscessus*
G150	0.063	0.063	*subsp. abscessus*
G151	0.063	0.063	*subsp. abscessus*
G152	0.125	0.063	*subsp. abscessus*
G153	0.063	0.063	*subsp. massiliense*
G155	0.063	0.063	*subsp. abscessus*
G156	0.125	0.125	*subsp. massiliense*
G157	0.125	0.063	*subsp. massiliense*
G158	0.063	0.063	*subsp. abscessus*
G159	0.063	0.063	*subsp. abscessus*
G160	0.125	0.063	*subsp. abscessus*
G161	0.063	0.063	*subsp. abscessus*
G162	0.063	0.063	*subsp. abscessus*
G163	0.125	0.125	*subsp. abscessus*
G164	0.063	0.032	*subsp. abscessus*
G165	0.063	0.063	*subsp. abscessus*
G169	0.063	0.063	*subsp. abscessus*
G170	0.063	0.063	*subsp. abscessus*
G172	0.063	0.063	*subsp. abscessus*
G173	0.125	0.125	*subsp. massiliense*
G174	0.063	0.063	*subsp. abscessus*
G175	0.25	0.125	*subsp. abscessus*
G176	0.063	0.063	*subsp. abscessus*
G177	0.063	0.063	*subsp. abscessus*
G178	0.063	0.063	*subsp. abscessus*
G179	0.063	0.063	*subsp. abscessus*
G180	0.063	0.063	*subsp. abscessus*
G181	0.063	0.063	*subsp. abscessus*
G182	0.063	0.063	*subsp. abscessus*
G183	0.063	0.063	*subsp. abscessus*
G184	0.063	0.063	*subsp. abscessus*
G185	0.063	0.063	*subsp. abscessus*
G186	0.063	0.063	*subsp. abscessus*
G187	0.063	0.063	*subsp. abscessus*
G188	0.063	0.063	*subsp. massiliense*
G189	0.063	0.063	*subsp. massiliense*
G190	0.063	0.063	*subsp. massiliense*
G192	0.063	0.063	*subsp. abscessus*

G193	0.063	0.063	*subsp. abscessus*
G194	0.063	0.063	*subsp. abscessus*
G195	0.063	0.063	*subsp. abscessus*
G196	0.063	0.063	*subsp. abscessus*
G197	0.063	0.063	*subsp. abscessus*
G198	0.063	0.063	*subsp. abscessus*
G199	0.125	0.063	*subsp. massiliense*
G200	0.063	0.063	*subsp. abscessus*
G201	0.125	0.063	*subsp. abscessus*
G203	0.063	0.063	*subsp. massiliense*
G204	0.063	0.063	*subsp. abscessus*
G205	0.063	0.063	*subsp. abscessus*
G206	0.063	0.063	*subsp. abscessus*
G207	0.063	0.063	*subsp. massiliense*
G208	0.125	0.125	*subsp. abscessus*
G210	0.063	0.063	*subsp. abscessus*
G211	0.063	0.063	*subsp. abscessus*
G213	0.063	0.063	*subsp. abscessus*
G215	0.063	0.063	*subsp. abscessus*
G216	0.063	0.063	*subsp. abscessus*
G218	0.063	0.063	*subsp. abscessus*
G219	0.063	0.063	*subsp. abscessus*
G220	0.063	0.063	*subsp. massiliense*

Table S2. MICs of MRX-6038 and GSK656 for other NTM clinical isolates

Isolate	MICs of MRX-6038 (mg/L)	MICs of GSK656 (mg/L)	species
1	2	>128	*M. intracellulare*
2	2	>128	*M. intracellulare*
3	2	>128	*M. intracellulare*
4	4	>128	*M. intracellulare*
5	4	>128	*M. intracellulare*
6	4	>128	*M. intracellulare*
7	4	>128	*M. intracellulare*
8	4	>128	*M. intracellulare*
9	4	>128	*M. intracellulare*
10	4	>128	*M. intracellulare*
11	4	>128	*M. intracellulare*
12	4	>128	*M. intracellulare*
13	4	>128	*M. intracellulare*
14	8	>128	*M. intracellulare*
15	>8	>128	*M. intracellulare*
1	0.5	>128	*M. avium*
2	0.5	>128	*M. avium*
3	1	>128	*M. avium*
4	1	>128	*M. avium*
5	1	>128	*M. avium*
6	1	>128	*M. avium*
7	2	>128	*M. avium*
8	2	>128	*M. avium*
9	4	>128	*M. avium*
10	4	>128	*M. avium*
11	4	>128	*M. avium*
12	4	>128	*M. avium*
13	8	>128	*M. avium*
14	8	>128	*M. avium*
1	0.125	>8	*M. fortuitum*
2	0.125	>8	*M. fortuitum*
3	0.125	>8	*M. fortuitum*
4	0.125	>8	*M. fortuitum*

Table S3. MICs and MBCs of GSK656 and MRX-6038 for *M. abscessus*

Isolate	GSK656		MRX-6038	
	MIC (mg/L)	MBC (mg/L)*	MIC (mg/L)	MBC (mg/L)
A215	0.063	2	0.063	0.5
A217	0.063	2	0.063	0.5
A232	0.063	1	0.063	0.125
A274	0.125	>4	0.125	2
A35	0.063	>2	0.063	0.25
G73	0.063	>2	0.063	1
G164	0.063	>2	0.063	0.25
A51	0.063	>2	0.063	0.25
A222	0.063	>2	0.063	0.25
G74	0.063	>4	0.063	1
A311	0.063	1	0.063	0.25
G104	0.063	2	0.125	0.25
A213	0.063	2	0.125	0.25
A353	0.125	2	0.125	0.25
A255	0.063	2	0.063	0.25
G87	0.063	>2	0.063	0.5
G182	0.063	>2	0.063	1
G110	0.063	>2	0.063	1
A197	0.063	>2	0.063	1
A268	0.125	>4	0.125	2

*Values >2 mg/L and >4 mg/L indicate a MBC value greater than the highest GSK656 concentration tested.

Table S4. Comparison of MRX-5 and macrolides MIC values effective in treating *M. abscessus*

Isolate or reference strain	Subspecies	MIC of the drug indicated (mg/L)		
		MRX-5	CLA*	AZM*
A217	*abscessus*	0.125	1	8
A27	*abscessus*	0.125	1	16
G164	*abscessus*	0.125	2	32
A222	*massiliense*	0.250	1	16
A268	*massiliense*	0.125	0.5	16
G74	*massiliense*	0.125	2	16
ATCC 19977	*abscessus*	0.125	0.5	4
CIP108297	*massiliense*	0.125	0.5	4

*CLA, clarithromycin; AZM, azithromycin.

Table S5. Toxicity of in mice administered with MRX-6038

Group	Day / Weight (g)				14 days blood chemistry (U/L)		
	Day 1	Day 7	Day 14	Day14-Day1	ALT*	AST*	ALP*
Blank	195.7±8.9	218±8.9	235.3±16.0	39.7	42.5±4.5	132.5±7.1	195±27.1
Vehicle	191.3±5.4	211±10.0	220.5±14.5	29.2	49.2±8.3	173.2±44.3	234±94.9
40 mg/kg/day	196.2±11.1	216.3±8.9	230.3±12.8	34.2	53±9.6	231.7±101.0	222±24.1
80 mg/kg/day	189.3±6.2	208.3±8.2	217.3±10.2	28.0	40.5±2.3	149.7±13.3	195±42.5
160 mg/kg/day	197.8±6.9	216.7±11.2	232.7±13.8	34.8	39.5±5.2	145.2±10.9	180.8±40.4

*****ALT: alanine aminotransferase; AST: aspartate aminotransferase, ALP: alkaline phosphatase.

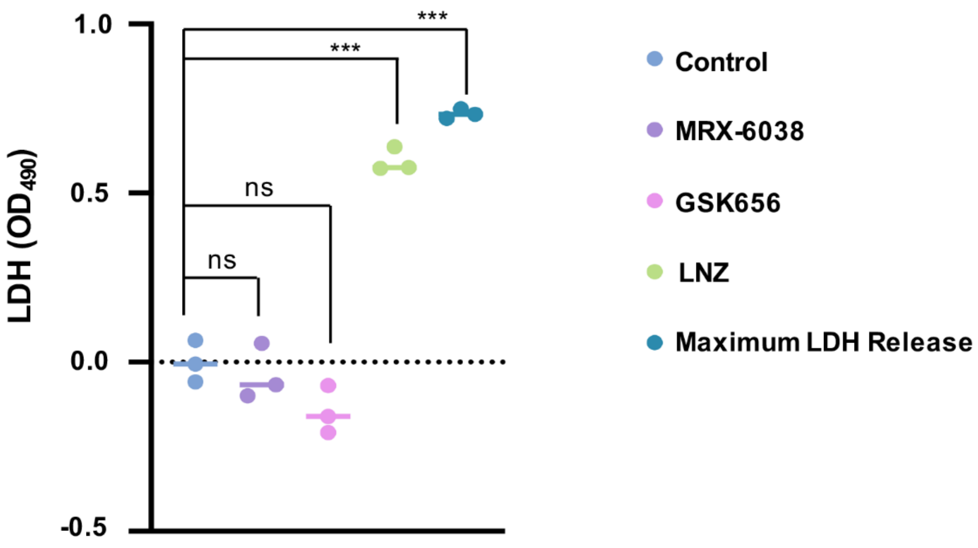

Figure S1: Cell cytotoxicity of MRX-6038 and GSK656. The data are LDH ± SD; n=3. LNZ, linezolid. LDH, lactate dehydrogenase. Significantly different: ns, no significance; ****P* <0.001 (Student's *t*-test).
